# Association between Vitamin D Supplementation and Mental Health in Healthy Adults: A Systematic Review

**DOI:** 10.3390/jcm10215156

**Published:** 2021-11-03

**Authors:** Dominika Guzek, Aleksandra Kołota, Katarzyna Lachowicz, Dominika Skolmowska, Małgorzata Stachoń, Dominika Głąbska

**Affiliations:** 1 Department of Food Market and Consumer Research, Institute of Human Nutrition Sciences, Warsaw University of Life Sciences (WULS-SGGW), 159C Nowoursynowska Street, 02-776 Warsaw, Poland; 2 Department of Dietetics, Institute of Human Nutrition Sciences, Warsaw University of Life Sciences (WULS-SGGW), 159C Nowoursynowska Street, 02-776 Warsaw, Poland; aleksandra_kolota@sggw.edu.pl (A.K.); katarzyna_lachowicz@sggw.edu.pl (K.L.); dominika_skolmowska@sggw.edu.pl (D.S.); malgorzata_stachon@sggw.edu.pl (M.S.); dominika_glabska@sggw.edu.pl (D.G.)

**Keywords:** supplementation, supplement, vitamin D, cholecalciferol, mental health, depression, depressive symptoms, anxiety, mood, well-being, quality of life

## Abstract

Vitamin D is considered to be a crucial factor that influences symptoms of depression, negative emotions, and quality of life, but to date, no systematic review has been conducted with regard to its effect on other domains of mental health. The aim of the study was to evaluate the influence of vitamin D supplementation on mental health in healthy adults. The systematic review was registered in the PROSPERO database (CRD42020155779) and performed according to the PRISMA guidelines. The literature search was conducted in PubMed and Web of Science databases and included intervention studies published until October 2019. The human studies were included if the supplementation regimen involved the administration of a specified dosage of vitamin D to an adult sample. A total of 7613 records were screened and assessed independently by two researchers, based on their title, abstract, and full text sequentially. Finally, 14 studies were included, and their risk of bias was assessed using the Newcastle–Ottawa Scale (NOS). The studies were included if they presented the results of various doses of vitamin D, compared the supplementation results with the placebo effect, compared the outcome with no supplementation, or observed effect of specific dose applied. The assessed mental health outcomes mainly included depressive symptoms, or depression, well-being, quality of life, mood, general mental component, and anxiety, but single studies also included other parameters such as distress, impression of improvement, and fear of falling and flourishing. The results of the majority of studies did not confirm a positive influence of vitamin D supplementation. None of the high-quality studies (assessed using NOS), which evaluated outcomes other than depression, supported the hypothesis that vitamin D supplementation effectively ameliorates mental health issues, while they present conflicting evidence for depression. Some studies indicated that supplementation should be combined with physical activity to provide effective results, and that supplementation is less effective than vitamin D supply from food sources. The included studies were conducted in diverse populations and followed various doses and intervals of administration, so the results may be incomparable, which should be considered as a limitation. The conducted systematic review did not provide strong evidence for a positive effect of vitamin D supplementation on mental health in healthy adults.

## 1. Introduction

Vitamin D is considered to be a crucial nutrient for calcium absorption and homeostasis, thus influencing bone health and metabolism [1]. However, several investigations in the last few decades have revealed that this vitamin is associated with numerous extra-skeletal effects [2] and that it plays a pivotal role in the prevention and treatment of multiple diseases [3]. Taking this into account, the serious public health problem appeared, as vitamin D insufficiency is estimated to affect about 50% of the global population, and vitamin D deficiency affects 1 billion people, independent of their age and ethnicity [4]. This problem was addressed in a prominent meta-analysis conducted by Garland et al. [5], which demonstrated that low serum levels of 25-hydroxyvitamin D (25(OH)D) are associated with an increased rate of all-cause mortality. This finding prompted other authors to provide recommendations to protect the global population from deficiency by increasing the recommended vitamin D intake levels [6], as well as applying fortified products and through supplementation [7].

The results of prospective clinical trials presenting the effects of vitamin D supplementation are promising, indicating that it is a valuable nutrient, especially for individuals who are unable to meet the recommended dietary intake levels and are unable to receive an adequate amount of sunlight [8]. The most important results indicated that combined supplementation with calcium and vitamin D reduces the risk of total cancer, breast cancer, and colorectal cancer, which was found in Women’s Health Initiative (WHI) [9]. Similarly, a population-based, double-blind, randomized placebo-controlled trial by Lappe et al. [10] reported that combined calcium and vitamin D supplementation reduces all-cancer risk in postmenopausal women. At the same time, a systematic review and meta-analysis of randomized controlled trials by Jolliffe et al. [11] showed that vitamin D supplementation reduces the rate of moderate-to-severe chronic obstructive pulmonary disease (COPD) exacerbations in patients with low baseline 25(OH)D levels. Some studies indicated that vitamin D supplementation may reduce the risk of infection by SARS-CoV-2 and, thus, coronavirus-19 disease (COVID-19) and may also complement the applied treatment, but more studies are necessary to consolidate this theory [12]. Vitamin D supplementation may be needed to obtain and maintain the desirable blood levels of 25(OH)D [13], but the specific doses depend on the target group [14].

Vitamin D is found to be important not only for physical health problems but also to address various mental health issues, as suggested by some meta-analyses conducted mainly for depression. The meta-analyses by Vellekkatt and Menon [15], Shaffer et al. [16], and Spedding [17], concluded that vitamin D supplementation may effectively alleviate the symptoms of depression. However, the results are not consistent, as the meta-analyses by Gowda et al. [18] and Li et al. [19] presented contradictory results and reported that vitamin D does not improve the symptoms of depression, which may be explained by the fact that the studies in this meta-analyses included individuals with low levels of depression symptoms and adequate baseline 25(OH)D levels [18], as well as the publications reviewed were characterized by a high risk of bias [19]. The positive effect of vitamin D supplementation was also associated with a reduction in the occurrence of negative emotions, as indicated in the meta-analysis by Cheng et al. [20], and for improvement of quality of life, as indicated in the systematic review by Hoffmann et al. [21].

Despite the fact that some systematic reviews and meta-analyses evaluated the influence of vitamin D supplementation on some aspects of mental health, namely, symptoms of depression [15,16,17,18,19], negative emotions [20], and quality of life [21], its effect on other mental health problems has not been investigated so far, although some single studies addressed this issue. Therefore, the aim of the present study was to evaluate the influence of vitamin D supplementation on mental health in healthy adults.

## 2. Materials and Methods

### 2.1. The Registration and Design

The systematic review was registered in the International Prospective Register of Systematic Reviews (PROSPERO) database (CRD42020155779), within the common registration with the previous studies, which covered the influence of vitamin D on mental health in healthy children [22], as well as in adults with diabetes [23] and in adults with inflammatory bowel diseases and irritable bowel syndrome [24]. The systematic review was based on the Preferred Reporting Items for Systematic Reviews and Meta-Analyses (PRISMA) guidelines [25] for the systematic literature search, screening, inclusion, and reporting. The literature search was conducted based on PubMed and Web of Science databases, and it included intervention studies published until October 2019, as the search procedure was conducted in October 2019.

### 2.2. The Eligibility and Inclusion

The eligible studies were to present the intervention including vitamin D supplementation and its influence on mental health outcomes. Only studies published in English, in a peer-reviewed journal were allowed.

The inclusion criteria were formulated as follows:

(1) Studied adult population;
(2) Applied vitamin D supplementation of the specified dose;
(3) Outcome including any mental health aspect assessed based on any method (including both subjective questionnaire and medical diagnosis).

The exclusion criteria were formulated as follows:

(1) Studies conducted in animal models;
(2) Studies conducted in a specific populations of individuals with any specific physical health problems (any physical symptom, disease, or disorder defining the studied group);
(3) Studies conducted in subjects with intellectual disabilities;
(4) Studies conducted in subjects with eating disorders;
(5) Studies conducted in subjects with neurological disorders (e.g., Alzheimer’s disease, epilepsy).
(6) Studies assessing influence of combined multiple nutrients supplemented;
(7) Studies assessing influence of maternal vitamin D supplementation on mental health in offspring.

No additional criteria, associated with the country, or population were formulated, and the patient, intervention/exposure, comparator, outcome, and study design (PICOS) criteria for inclusion and exclusion of studies are summarized in Table 1.

### 2.3. The Search Strategy

The applied detailed electronic search strategy for databases of PubMed and Web of Science is presented in Supplementary Table S1.

**Figure 1:**
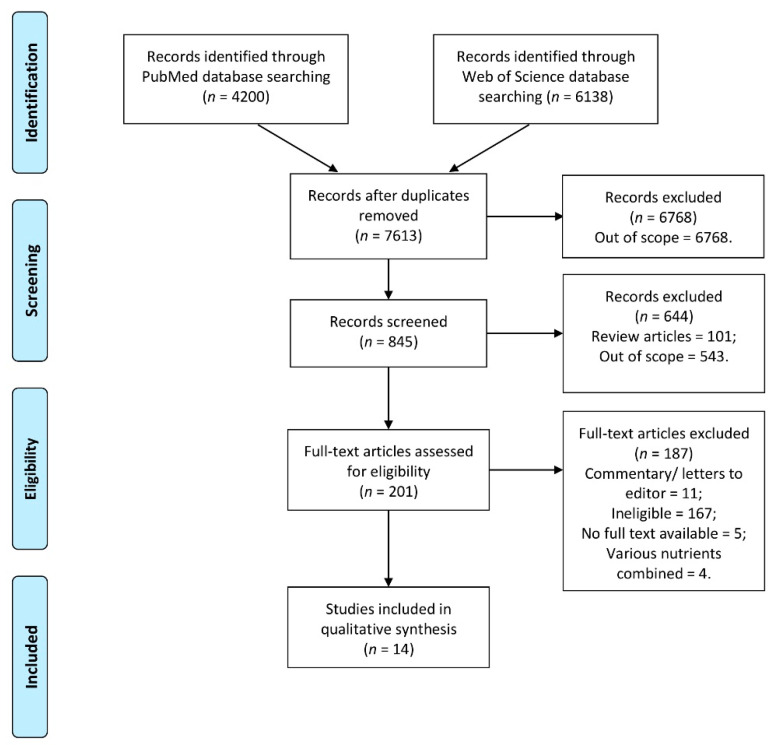
The inclusion procedure to the systematic review developed based on PRISMA 2009 flow diagram [23].

The identified studies were verified to remove duplicates, and they were assessed independently by 2 researchers, based on the title, to screen and assess studies for eligibility. This assessment was followed by the next step, which was conducted based on abstract, to assess studies for eligibility, which was also conducted independently by 2 researchers. If any disagreement appeared at any step, it was discussed with the other researcher. Afterwards, the full texts of the studies indicated as eligible were extracted, and if unavailable, the corresponding author was contacted to obtain full text. The full texts that were gathered were finally assessed by 2 researchers to confirm including them to a systematic review. If any disagreement appeared, it was discussed with the other researcher. The inclusion procedure is presented in Figure 1.

### 2.4. Procedure of Data Extraction

The procedure of data extraction was conducted independently by 2 researchers. If any information was unavailable in the article, the corresponding author was contacted to request additional information (referred to in the Results section as data provided on request). If any disagreement appeared, it was discussed with the other researcher. The extracted information included: study design and basic details of the studies included in the systematic review (country/location, studied group, time); characteristics of the groups studied (number of participants and of female participants, age, inclusion criteria/exclusion criteria); characteristics of the exposure and outcome studied (vitamin D measure, applied vitamin D supplementation, mental health outcome, psychological measure), and findings formulated (observations and conclusions as formulated by authors of the study).

According to the procedure recommended for the systematic reviews, the risk of bias resulting from methodological quality [26] was assessed while using the Newcastle–Ottawa Scale (NOS) [27]. The included studies were assessed for the selection (score of 0 to 4), comparability (score of 0 to 2), and exposure/outcome (score of 0 to 3). The final assessment was made within the following categories: very high risk of bias (total score of 0 to 3), high risk of bias (total score of 4 to 6), and low risk of bias (total score of 7 to 9) [28].

## 3. Results

The study design and basic details of the studies included in the systematic review [29,30,31,32,33,34,35,36,37,38,39,40,41,42] are presented in Table 2. The included studies were conducted mainly in the United States of America [30,31], Norway [33,38], and Poland [40,43], while single studies were conducted also in Canada [29], Australia [32], United Kingdom [34], Finland [35], Iran [36], New Zealand [37], Netherlands [40], and Switzerland [41]. A number of studies were conducted in community-based samples, but some of them included specific groups of outpatients visiting an endocrinology clinic [29], patients with vitamin D deficiency or low 25(OH)D blood levels [30,33], patients with a prior fall [35,41], patients with functional limitations [40], pregnant women under prenatal care [36], marathon runners [39], and participants of a training program [42].

The characteristics of the groups studied within the studies included in the systematic review is presented in Table 3. The included intervention studies were conducted mainly in small samples of respondents—less than 100 respondents [29,30,34,39,42]—or medium ones—less than 500 [33,35,36,37,38,40,41]—but two studies were conducted in large samples involving over 2000 [32] and over 80,000 participants [31]. The inclusion criteria mainly included the 25(OH)D blood level [29,30,32,33,38,40,42], but in some studies, history of falls was also taken into account [34,35,41].

The characteristics of the exposure and outcome studied within the studies included in the systematic review are presented in Table 4. The intervention studies included in the present review evaluated the effects of various doses of vitamin D [29,41], compared the supplementation results with the placebo effect [32,33,35,36,37,38,39,40], compared the outcome with no supplementation [31], or observed the effect of a specific dose applied [30,34,42]. In the majority of studies, vitamin D was applied for a period of a few months (6 months or shorter) [29,30,33,34,36,37,38,42], while in some, the supplementation protocol extended for one year [40,41] or even longer [31,32,35]; but, in one study, vitamin D was administered for only 2 weeks [39]. The assessed mental health outcomes included mainly depressive symptoms, or depression [30,31,33,35,36,37,38,40,41,42], well-being [29,32,35], quality of life [35,40,42], mood [29,37,39], general mental component [32,34,41], and anxiety [37,40]. However, single studies also evaluated other parameters such as distress [32], impression of improvement [32], and fear of falling [35] and flourishing [37].

The summary of concluded association between vitamin D supplementation and mental health in adults, accompanied by the Newcastle–Ottawa Scale (NOS) total score for the studies included in the systematic review, is presented in Table 5. The findings for the studies included in the systematic review are presented in Supplementary Table S2. Among the included studies, seven were associated with a low risk of bias (NOS score of 7–9) [28], some reported protective effects of vitamin D [31,36], and some found no beneficial effects for this vitamin [32,35,37,38,40]. However, it should be noted that the majority of the studies did not provide strong evidence for a positive influence of vitamin D supplementation. Moreover, none of the high-quality studies (associated with low risk of bias), which assessed outcomes other than depression (mental well-being [32,35], quality of life/health-related quality of life [35,40], anxiety [37], fear of falling [35], flourishing [37], mood [37]) supported positive effects of vitamin D supplementation. On the contrary, high-quality studies provided conflicting evidence for depression/depressive symptoms—either positive influence of vitamin D supplementation [30,36] or no such influence [37,38,40].

## 4. Discussion

The described inconsistent observations with regard to the effect of vitamin D supplementation on mental health are in agreement with the general controversies associated with the therapeutic use of vitamin D supplementation [43]. They are associated with no consistent recommendations of vitamin D intake [44], which for adults varies from 5 µg in Australia and New Zealand [45] to 20 µg in Germany, Austria, and Switzerland [46]. Similarly, there are also diverse opinions with regard to baseline levels of 25(OH)D in the serum, below which vitamin D deficiency occurs [47]. A threshold level of 30 nmol/L is specified by the United States (US) Institute of Medicine (IoM) [48] and the United Kingdom National Osteoporosis Society [49] and a level of 50 nmol/L by the US Endocrine Society [50] and European Food Safety Authority (EFSA) [51]. In addition, the vitamin D supplementation doses recommended by various authorities for the general population of adults also differ [52], ranging from daily supplementation limit of no more than 15 µg, recommended by IoM [53], to the maximum permissible level of 50 µg, recommended by US Endocrine Society [50].

The above-mentioned results show that little is known about the target 25(OH)D blood level, but also various recommendations of supplementation may be formulated, which in the studies included in the present systematic review ranged from 15 µg per day for at least 2 months [29] to 250 µg per day for 2 weeks [39]. Furthermore, some researchers did not apply daily vitamin D doses, but weekly [33,34,38], monthly [37,41], or even yearly doses [32]. However, the efficacy of vitamin D supplementation is dependent on the applied dosage [54] and time intervals proposed for this supplementation [55], so the results of the included studies may be incomparable. In addition, some countries have their national vitamin D fortification policy, which includes fortification of products such as milk (Finland, Sweden, and Iceland); nondairy milk alternatives, such as soy, rice, and oat drinks (Finland); fat spreads/margarines (Finland and United Kingdom) [56]. These products influence the vitamin D status in these populations and may also contribute to the differences in results between studies. However, if the dietary intake of vitamin D is not recorded within the study, it might not have been taken into account.

Bearing this in mind, it must be emphasized that the studies included in the systematic review were conducted in various populations, as well as various doses and intervals were followed for supplementation, so the results may have been incomparable, which is a limitation of the present analysis. Moreover, only studies published in English, in peer-reviewed journals were included, which means that some interesting results may not have been taken into account. The other issue is associated with the diverse quality of the included studies accompanied by the relatively low number of studies randomized against placebo, which may be associated with the risk of bias. Last but not least, the presented study included articles published until October 2019, while the further ones were not screened, which also should be indicated as one of the limitations.

As described above, the results of the conducted systematic review did not provide explicit evidence to demonstrate a positive effect of vitamin D supplementation on mental health in a population of adults. Not only did the majority of the included studies not support it, but also, many reported inconclusive results. Only 36% of the studies were classified as supporting [29,30,31,36,42], and among them, some suggested that specific conditions need to be met in order to observe the positive influence of vitamin D supplementation. One study confirmed that vitamin D supplementation may be beneficial to overcome depression only when it is combined with physical activity [42], while another study recommended the dietary intake primarily through food sources that provided better results, while for supplementation, less consistent results were documented [31]. Considering these findings, it may be concluded that vitamin D supplementation may be not so effective as dietary intake, but also that it should be an element of a broader treatment program, also including behavioral therapy. In spite of this, it is generally stated that if taking dietary supplements to correct vitamin D deficiency improves mental well-being, it would be a simple and cost-effective solution for patients at risk of depression and possibly other mental disorders [57]. However, the results of the conducted systematic review indicate that this solution may not be as effective as expected.

Although a majority of the high-quality studies indicate that vitamin D supplementation may have a positive impact on depression, other studies do not support the positive influence of vitamin D supplementation on other mental health problems. Therefore, vitamin D supplementation should not be treated as the only dietary intervention to be applied in the prevention and therapy of mental health disorders. The treatment protocol should not only include a broader dietary intervention, to ensure that an adequate amount of the vitamin D is obtained through the food sources, but should also include other dietary modifications. Such a modification should be based on the well-known association between diet and mental health, as indicated in the previous systematic reviews that determined the effects of fruit and vegetable intake [58] and various dietary patterns [59]. Moreover, physical activity should also be promoted as the confirmed factor associated with mental health [60], additionally supporting the positive influence of vitamin D.

## 5. Conclusions

The conducted systematic review did not confirm a positive influence of vitamin D supplementation on mental health in healthy adults. It was supported only by some studies, which mainly included depression and not other mental health problems. Some studies indicated that this supplementation should be combined with physical activity to produce effective results, while some studies revealed that supplementation is less effective than vitamin D supply from food sources.

## Figures and Tables

**Table 1:** Patient, intervention/exposure, comparator, outcome, study design (PICOS) criteria for inclusion and exclusion of studies.

Parameter	Inclusion Criteria	Exclusion Criteria
Population	Healthy adults	Children and adolescents, individuals with any specific physical health problems, intellectual disabilities, eating disorders, or neurological disorders
Intervention/exposure	Participants assessed during vitamin D supplementation	Combined multiple nutrients supplemented
Comparison	Influence on a mental health outcomes assessed while compared with baseline/placebo/various doses and regimens	Lack of comparison
Outcome	Any aspect of mental health associated with any area of the broad spectrum of general mental health	Patients assessed for cognitive function
Study design	Peer-reviewed articles published in English, including: randomized controlled trials, randomized crossover trials, cohort studies, case-control studies, and cross-sectional studies	Articles not published in English, reviews, meta-analyses, expert opinions, letters to editor, comments, studies in animal models, methodological articles, case reports, and conference reports

**Table 2:** The study design and basic details of the studies included in the systematic review.

Ref.	Authors, Year	Study Design	Country/Location	Studied Group	Time
[29]	Vieth et al., 2004	Blinded, randomized trial	Canada/Toronto	Outpatients of endocrinology clinic with 25(OH)D < 61 nmol/L (Study 1)	Winter 2001–2002 (Study 1)
				Outpatients of endocrinology clinic with 25(OH)D < 51 nmol/L (Study 2)	Winter 2002–2003 (Study 2)
[30]	Shipowick et al., 2009	Quasi-experimental pilot study	United States of America/Washington	Female patients treated at a medical clinic for vitamin D deficiency or insufficiency	Not specified
[31]	Bertone-Johnson et al., 2011	Cross-sectional prospective analysis within Women’s Health Initiative Observational Study (WHI OS)	United States of America	Women aged 50–79 participating in WHI OS	1993–1998
[32]	Sanders et al., 2011	Double-blind, randomized, placebo-controlled trial within Vital D study	Australia/Barwon and Mornington Peninsula regions	Community-dwelling women aged at least 70 years participating in Vital D study	2005–2008
[33]	Kjaergaard et al., 2012	Nested case-control study and randomized clinical trial	Norway/Tromsø	Adults 30–75 years old with serum 25(OH)D levels below the 20 percentile (55 nmol/L) or above the 75 percentile (70 nmol/L) from the sixth Tromsø study	October 2009–November 2010
[34]	Cheema and Chaudhry, 2016	Prospective study	United Kingdom	Patients admitted with a fall with or without sustaining a fragility fracture post fall	Not specified
[35]	Patil et al., 2016	Randomized double-blind, placebo-controlled intervention trial with vitamin D and exercise (DEX trial)	Finland	Older Finnish women participating in DEX trial	2010–2013
[36]	Vaziri et al., 2016	Randomized controlled trial	Iran/Shiraz	Pregnant women under prenatal care in Hafez hospital	November 2014–October 2015
[37]	Choukri et al., 2018	Double-blind, placebo-controlled, randomized clinical trial	New Zealand/Dunedin	Healthy women aged 18–40 years	February–October 2013
[38]	Jorde and Kubiak, 2018	Randomized controlled trial within Tromsø study population	Norway/Tromsø	Adults aged 40–80 years participating in Tromsø Study	2015–2016
[39]	Krokosz et al., 2018	Double-blind, placebo-controlled, randomized study	Poland	Experienced marathon and ultramarathon male runners (aged 31–50 years) taking part in a 100 km track run	Not specified
[40]	de Koning et al., 2019	Randomized placebo-controlled clinical trial within D-Vitaal Study	Netherlands/Amsterdam	Community-dwelling older individuals with functional limitations participating in D-Vitaal Study	2013–2016
[41]	Gugger et al., 2019	1-year double-blind randomized clinical trial	Switzerland/Zurich	Community-dwelling older adults with a prior low-trauma fall in the previous year	January 2010–May 2011
[42]	Lipowski et al., 2019	Intervention program	Poland/Gdansk	Elderly women participating in a Nordic walking training program	October 2018 *

* data provided on request. 25-hydroxyvitamin D (25(OH)D).

**Table 3:** The characteristics of the groups studied within the studies included in the systematic review.

Ref.	Authors, Year	Number of Participants (Number of Female Participants)	Age (Mean with SD/Range)	Inclusion Criteria/Exclusion Criteria
[29]	Vieth et al., 2004	64 (53) (Study 1) 112 (87) (Study 2)	Mean of 48–56 years depending on the sub-group	Inclusion: outpatients of endocrinology clinic; low 25(OH)D that demonstrated a need for supplementation Exclusion: not specified
[30]	Shipowick et al., 2009	6 (6)	42.2 ± 13.2 years	Inclusion: female patients; serum 25(OH)D levels below 100 nmol/L Exclusion: mental impairments; dementia; language barriers; using or planned to use tanning beds or other phototherapy; planned on traveling to sunnier, more tropical areas during the winter; taking or planning to take antidepressants
[31]	Bertone-Johnson et al., 2011	81,189 (81,189)	50–79 years at baseline	Inclusion: female participants of WHI OS Exclusion: enrolment in a WHI clinical trial; medical conditions likely to result in death within 3 years; previous history of cancers (except nonmelanoma skin cancer); conditions that were likely to interfere with retention in the study; implausible calorie intake (<600 and >5000 kcal/day); missing data
[32]	Sanders et al., 2011	2258 (2258)	At least 70 years at baseline	Inclusion: female participants of Vital D study; aged at least 70 years at baseline; identified risk factor for hip fracture (maternal hip fracture, self-reported “faller”, fracture since aged 50), and/or high risk of low vitamin D and osteoporosis Exclusion: inability to provide informed consent or falls/fracture data; permanent resident of high-level care facility; albumin-corrected calcium 42.65 mmol/L; vitamin D supplementation 110 µg; other parameters relating to bone health
[33]	Kjaergaard et al., 2012	357 (179)	53.6 ± 10.3 years for case group 55.1 ± 9.4 years for control group	Inclusion: participants of the sixth Tromsø study; 30–75 years, serum 25(OH)D levels below the 20 percentile (55 nmol/L) or above the 75 percentile (70 nmol/L) Exclusion: history of known diabetes, coronary heart disease, or stroke in the past 12 months; cancer; kidney stones; any conditions needing medical attention; possible primary hyperparathyroidism (PTH > 5.0 pmol/L combined with serum calcium > 2.50 mmol/L); males with serum creatinine > 130 mmol/L and females with serum creatinine > 110 mmol/L; systolic blood pressure > 174 mmHg or diastolic blood pressure > 104 mmHg; pregnant or lactating women; fertile women below the age of 50 years without adequate contraception; reported use of vitamin D supplements, antidepressants, or other mood stabilizing medication; regular use of a solarium; planned trip to a sunny location in the trial period; Beck Depression Inventory (BDI) score > 29; Montgomery–Åsberg Depression Rating Scale (MADRS) score >34; serious depression in the Structured Clinical Interview for DSM-IV Axis I Disorders–Clinician Version (SCID-CV)
[34]	Cheema and Chaudhry, 2016	38 (20)	80.2 ± 12.0 years	Inclusion: patients admitted with falls Exclusion: not specified
[35]	Patil et al., 2016	409 (409)	Mean of 73–75 years depending on the sub-group	Inclusion: participants of the DEX trial; 70–80 years; home-dwelling women; fallen at least once during the previous 12 months; no contraindications to exercise Exclusion: regular use of vitamin D supplements; moderate to vigorous exercise > 2 h per week
[36]	Vaziri et al., 2016	169 (169)	26.3 ± 4.6 years	Inclusion: ≥ 18 years; women; healthy—no history of mental illness and internal diseases such as hyper/hypothyroidism, parathyroid, renal, diabetes, and heart diseases; no addiction to any kind of narcotic drugs; living with a husband; a singleton live fetus; without any pregnancy complications such as preeclampsia, gestational diabetes, ruptured membranes, and suspicion of preterm birth; no previous cesarean sections; gestational age of 26–28 weeks based on ultrasound results; Edinburgh Postnatal Depression Scale (EPDS) baseline scores of 0–13 Exclusion: not providing blood sample at the onset of the study; less than 8 weeks consumption of vitamin D3 supplementations; irregular consumption of vitamin D3 supplementations
[37]	Choukri et al., 2018	150 (150)	24.2 ± 6.0 years	Inclusion: 18–40 years; women; not currently pregnant or breastfeeding; access to the Internet; willing to provide a repeated blood sample Exclusion: current/planned vitamin D supplementation (including as part of a multivitamin supplement); chronic liver and kidney disease; arteriosclerosis or cardiac function impairment; sarcoidosis and other possible granulomatous diseases; medication, including anticonvulsants, glucocorticoids, and barbiturates that might affect vitamin D metabolism; overseas travel during the study period
[38]	Jorde and Kubiak, 2018	408 (191)	52.0 ± 8.8 years	Inclusion: age 40–80 years; vitamin D insufficiency (serum 25(OH)D < 42 nmol/L) Exclusion: granulomatous diseases; diabetes; renal stones in the last 5 years; serious diseases making the subject unfit for participation; vitamin D supplementation of > 20 μg per day; use of solarium on a regular basis; planned holiday in tropical areas during the study period; women of childbearing potential without use of acceptable contraception
[39]	Krokosz et al., 2018	20 (0)	40.7 ± 7.1 years	Inclusion: male; aged 31–50 years; experienced marathon and ultramarathon runners taking part in a 100 km track run Exclusion: not specified
[40]	de Koning et al., 2019	155 (89)	68.4 ± 5.3 years *	Inclusion: age 60–80 years; presence of depressive symptoms (Center of Epidemiological Studies–Depression scale (CES–D) score of ≥16); ≥1 functional limitation (e.g., difficulties with walking, climbing stairs, or dressing oneself); serum 25(OH)D concentration 15–50 nmol/L in winter or 15–70 nmol/L in summer Exclusion: current major depressive disorder diagnosis; life-threatening illness; current antidepressant medication; vitamin D supplementation of >10 μg per day; calcium supplementation of >1000 mg per day
[41]	Gugger et al., 2019	200 (134)	78 (71–92) years	Inclusion: age ≥ 70 years; pre-frail (low-trauma fall in the previous 12 months); Mini-Mental State Examination (MMSE) score of ≥27; a normal clock test Exclusion: unwillingness to stop additional vitamin D supplementation during the trial; insufficient mobility to come to the study center
[42]	Lipowski et al., 2019	52 (52)	69.8 ± 4.7 years	Inclusion: women; participating in a Nordic walking training program; baseline 25(OH)D3 concentration above 50 nmol/L Exclusion: uncontrolled hypertension (diastolic blood pressure over 100 mmHg); history of cardiac arrhythmia; cardio-respiratory disorders; orthopedic problems

* data provided on request.

**Table 4:** The characteristics of the exposure and outcome studied within the studies included in the systematic review.

Ref.	Authors, Year	Vitamin D Measure	Vitamin D Supplementation Dose and Regimen	Mental Health Outcome	Psychological Measure
[29]	Vieth et al., 2004	25(OH)D blood level 1,25(OH)2D blood level	15 µg vs. 100 µg/day for at least 2–6 months or > 6 months	(1) Energy and mood (2) Well-being	(1) Brief questionnaire, including 6 questions (energy level, mood, sleeping problems, lost interest or pleasure, ability to concentrate, changes of weight) (2) Brief questionnaire, including 10 questions (general health, feeling rested, down feeling/inappropriate guilt, feeling socially active, being indecisive, feeling productive and creative, appetite, cravings for carbohydrates, dealing with daily stress, feeling irritable or anxious)
[30]	Shipowick et al., 2009	25(OH)D blood level	125 µg/day for 2 months	Depression	Beck Depression Inventory–Second Edition (BDI-II)
[31]	Bertone-Johnson et al., 2011	No biochemical assessment —supplement use questionnaire	None vs. <10 µg vs. 10–20 µg vs. > 20 µg/day for 3 years based on the questionnaire retrospective data	Depressive symptoms	Burnam 8-item scale for depressive symptomsCurrent antidepressant medication use
[32]	Sanders et al., 2011	25(OH)D blood level	12,500 µg/year (as one dose) vs. placebo for 3–5 years	(1) Psychological distress (2) Mental component (3) Well-being (4) Impression of improvement	(1) General Health Questionnaire with 12 items (GHQ-12) (2) Short Form Health Survey (SF-12) (3) World Health Organization (WHO) Well-Being Index (4) Patient Global Impression of Improvement (PGI-I) scale
[33]	Kjaergaard et al., 2012	25(OH)D blood level	500 µg/week vs. placebo for 6 months	Depressive symptoms	(1) Beck Depression Inventory–Second Edition (BDI-II) (2) Hospital Anxiety and Depression Scale (HADS) (3) Seasonal Pattern Assessment Scale (SPAQ) (4) Montgomery–Åsberg Depression Rating Scale (MADRS) (5) Structured Clinical Interview for DSM-IV Axis I Disorders–Clinician Version (SCID-CV)
[34]	Cheema and Chaudhry, 2016	25(OH)D blood level	1500 µg/week for 2 months (followed by maintenance regimen of 10 µg and 600 mg of calcium)	Mental component	Short Form Health Survey (SF-12)
[35]	Patil et al., 2016	25(OH)D blood level	20 µg/day vs. placebo for 24 months	(1) Quality of life (2) Well-being (3) Fear of falling (4) Fear of falling	(1) Leipad questionnaire (2) World Health Organization (WHO) Well-Being Index (WHO-5) (3) Falls Efficacy Scale International (FES-I) (4) Single-Question Visual Analogue Scale (VAS)
[36]	Vaziri et al., 2016	25(OH)D blood level	50 µg/day vs. placebo from 26–28 weeks of gestation until childbirth	Depression	Edinburgh Postnatal Depression Scale (EPDS)
[37]	Choukri et al., 2018	25(OH)D blood level	1250 µg/month vs. placebo for 6 months	(1) Depressive symptoms (2) Anxiety (3) Flourishing (4) Positive and negative mood	(1) Center for Epidemiologic Studies Depression Scale (CES-D) (2) Hospital Anxiety and Depression Scale (HADS) (3) Flourishing Scale (FS) (4) Questions about 9 positive affects (happy, excited, cheerful, pleasant, calm, energetic, enthusiastic, content, relaxed) and 9 negative ones (nervous, dejected, irritable, hostile, sad, angry, unhappy, anxious, tense) across 3 consecutive days
[38]	Jorde and Kubiak, 2018	25(OH)D blood level	500 µg/week (following bolus dose of 2500 µg/day) vs. placebo for 4 months	Depression	Beck Depression Inventory–Second Edition (BDI-II)
[39]	Krokosz et al., 2018	25(OH)D blood level	250 µg/day vs. placebo for 2 weeks preceding the race	Mood states	University of Wales Institute of Science and Technology (UWIST) Mood Adjective Check List (UMACL) with Polish adaptation
[40]	de Koning et al., 2019	25(OH)D blood level	30 µg/day vs. placebo for 12 months	(1) Depressive symptoms (2) Health-related quality of life (HRQoL) (3) Anxiety	(1) Center for Epidemiologic Studies Depression Scale (CES-D) (2) EuroQol-5 Dimensions (EQ-5D) and Short Form–36 Health Survey (SF-36) (3) Beck Anxiety Inventory (BAI)
[41]	Gugger et al., 2019	25(OH)D blood level	600 µg vs. 1500 µg vs. 600 µg + 300 µg of calcifediol/month for 12 months	(1) Mental health (2) Depression	(1) Mental Component Summary (MCS) of Short Form–36 Health Survey (SF-36) (2) 15-Item Geriatric Depression Scale (GDS-15)
[42]	Lipowski et al., 2019	25(OH)D blood level	100 µg/day for 12 weeks	(1) Depressive symptoms (2) Health-related quality of life (HRQoL)	(1) Beck Depression Inventory–Second Edition (BDI-II) (2) Short Form–36 Health Survey (SF-36)

**Table 5:** The summary of concluded association between vitamin D supplementation and mental health in adults, accompanied by the Newcastle–Ottawa Scale (NOS) total score for the studies included in the systematic review.

Ref.	Authors, Year	Concluded Association between Vitamin D Supplementation and Mental Health in Adults		Quality ^b^
		Studied Outcome	Supporting/Inconclusive/Not Supporting ^a^	
[29]	Vieth et al., 2004	Well-being	Supporting	4
[30]	Shipowick et al., 2009	Depressive symptoms	Supporting	5
[31]	Bertone-Johnson et al., 2011	Depressive symptoms	Supporting, but less effective for supplementation than for food sources	8
[32]	Sanders et al., 2011	Mental well-being	Not supporting	7
[33]	Kjaergaard et al., 2012	Depressive symptoms	Not supporting	5
[34]	Cheema and Chaudhry, 2016	Mental component	Not supporting	5
[35]	Patil et al., 2016	Quality of life, fear of falling, mental well-being	Not supporting	9
[36]	Vaziri et al., 2016	Perinatal depression	Supporting	8
[37]	Choukri et al., 2018	Depression, anxiety, flourishing, mood	Not supporting	7
[38]	Jorde and Kubiak, 2018	Depressive symptoms	Not supporting	8
[39]	Krokosz et al., 2018	Mood	Inconclusive	3
[40]	de Koning et al., 2019	Depressive symptoms, health related quality of life	Not supporting	9
[41]	Gugger et al., 2019	Mental health	Inconclusive	4
[42]	Lipowski et al., 2019	Depressive symptoms	Supporting while combined with physical activity	5

^a^ Supporting—concluded positive influence of applied vitamin D supplementation on mental health; not supporting—concluded no positive influence of applied vitamin D supplementation on mental health; inconclusive—no clear association between applied vitamin D supplementation and mental health; ^b^ the Newcastle–Ottawa Scale (NOS) total score within the following categories: very high risk of bias (0–3 NOS points), high risk of bias (4–6 NOS points), and low risk of bias (7–9 NOS points) [28].

## Supplementary Materials

The following are available online at https://www.mdpi.com/article/10.3390/jcm10215156/s1. Supplementary Table S1: The applied detailed electronic search strategy for databases of PubMed and Web of Science, Supplementary Table S2: The findings for the studies included in the systematic review.
