# Supplementary material for: Association between Vitamin D Supplementation and Mental Health in Healthy Adults: A Systematic Review
Source: J Clin Med. 2021 Nov 3;10(21):5156. doi: 10.3390/jcm10215156 (PMC8584834; doi:10.3390/jcm10215156)
Supplement: Supplementary file 1 [file jcm-10-05156-s001.zip › jcm-1422279-supplementary.pdf]

Journal of
Clinical Medicine

MDPI

Systematic Review

Association between Vitamin D Supplementation and Mental Health in Adults: A Systematic Review

Dominika Guzek, Aleksandra Kołota, Katarzyna Lachowicz, Dominika Skolmowska, Małgorzata Stachoń, and Dominika Głąbska

Supplementary Table S1. The applied detailed electronic search strategy for databases of PubMed and Web of Science.

Database	Applied Full Electronic Search Strategy
PubMed	(((mental health[Title/Abstract] OR mental disorders[Title/Abstract] OR mental disorder[Title/Abstract] OR psychological distress[Title/Abstract] OR mood disorders[Title/Abstract] OR depression[Title/Abstract] OR suicidal[Title/Abstract] OR suicide[Title/Abstract] OR anxiety[Title/Abstract] OR well-being[Title/Abstract] OR wellbeing[Title/Abstract] OR quality of life[Title/Abstract] OR self esteem[Title/Abstract] OR self-esteem[Title/Abstract] OR self efficacy[Title/Abstract] OR self-efficacy[Title/Abstract] OR resilience[Title/Abstract] OR empowerment[Title/Abstract] OR social participation[Title/Abstract] OR mental capital[Title/Abstract] OR life skills[Title/Abstract] OR emotional[Title/Abstract] OR psychology[Title/Abstract] OR psychosocial[Title/Abstract] OR psychiatry[Title/Abstract])) AND (vitamin D[Title/Abstract] OR vitamin D2[Title/Abstract] OR vitaminD3[Title/Abstract] OR D2[Title/Abstract] OR D3[Title/Abstract] OR ergocalciferol[Title/Abstract] OR cholecalciferol[Title/Abstract] OR 25-hydroxyvitamin D[Title/Abstract] OR 3-epi-25hydroxyvitaminD[Title/Abstract] OR calcitriol[Title/Abstract] OR dihydroxycholecalciferol[Title/Abstract])) NOT (animal NOT (animal AND human)[MeSH Terms])
Web of Science	(TS=("vitamin D" OR "vitamin D2" OR "vitamin D3" OR "D2" OR "D3" OR "ergocalciferol" OR "cholecalciferol" OR "25-hydroxyvitamin D" OR "3-epi-25 hydroxyvitamin D" OR "calcitriol" OR "dihydroxycholecalciferol") AND TS=("mental health" OR "mental disorders" OR "mental disorder" OR "psychological distress" OR "mood disorder" OR "depression" OR "suicidal" OR "suicide" OR "anxiety" OR "well-being" OR "wellbeing" OR "quality of life" OR "self esteem" OR "self-esteem" OR "self efficacy" OR "self-efficacy" OR "resilience" OR "empowerment" OR "social participation" OR "mental capital" OR "life skills" OR "emotional" OR "psychology" OR "psychosocial" OR "psychiatry") NOT TS=("animal" NOT ("animal" AND "human")))

Supplementary Table S2. The findings formulated within the studies included in the systematic review.

Ref.	Observation	Conclusion
[27]	In Study 1, between December and February, wellbeing score improved more for the 100 μg/day group than for the lower-dosed group ($p = 0.036$). In Study 2, winter wellbeing scores improved with both doses of vitamin D ($p < 0.001$).	The work confirms the safety and efficacy of both 15 and 100 μg/day vitamin D in patients who needed additional vitamin D.
[28]	Vitamin D supplementation was associated not only with an increase in the serum 25(OH)D levels by an average of 27 ng/ml but also with a decline in the BDI-II scores of an average of 10 points.	The study suggests that supplemental vitamin D3 reduces depressive symptoms.
[29]	After age, physical activity, and other factors were controlled for, women who reported a total intake of ≥ 20 μg vitamin D/d had a prevalence OR for depressive symptoms of 0.79 (95% CI: 0.71, 0.89; *P*-trend <0.001) compared with women who reported a total intake of < 2.5 μg vitamin D/d. In analyses limited to women without evidence of depression at baseline, an intake of ≥ 10 μg compared with <2.5 μg vitamin D/day from food sources was associated with 20% lower risk of depressive symptoms at year 3 (OR: 0.80; 95% CI: 0.67, 0.95; *P*-trend = 0.001). The results for supplemental vitamin D were less consistent, as were the results from secondary analyses that included as cases women who were currently using antidepressant medications.	The findings support a potential inverse association of vitamin D intake, primarily from food sources, and depressive symptoms in postmenopausal women. Additional prospective studies and randomized trials are essential in establishing whether the improvement of vitamin D status holds promise for the prevention of depression, the treatment of depression, or both.
[30]	No significant differences between the vitamin D and placebo groups were detected in any of the measured outcomes of mental health. Serum 25-hydroxyvitamin D levels in the vitamin D group were 41% higher than the placebo group 12 months following their annual dose. Despite this difference, scores from the questionnaires did not differ. Furthermore, there was no interaction between those on anti-depressant/anxiety medication at baseline and the treatment groups.	The lack of improvement in indices of mental well-being in the vitamin D group does not support the hypothesis that an annual high dose of vitamin D3 is a practical intervention to prevent depressive symptoms in older community-dwelling women.
[31]	Participants with low 25(OH)D levels at baseline were more depressed than participants with high 25(OH)D levels. In the intervention study no significant effect of high-dose vitamin D was found on depressive symptom scores when compared with placebo.	Low levels of serum 25(OH)D are associated with depressive symptoms, but no effect was found with vitamin D supplementation.
[32]	After treatment, the mean difference of mental component score for the whole cohort was 1.05 (CI −2.6 to 4.7), $p = 0.56$.	Quality-of-life indicators did not improve.
[33]	When comparing with the placebo without exercise group, there were no statistically significant differences between groups receiving either vitamin D, exercise or both treatments for changes in quality of life or mental wellbeing (although a slight decline was seen in mental wellbeing in those receiving vitamin D only, $p = 0.044$). The initial slight reduction in fear of falling was significant in all intervention groups compared with controls ($p < 0.05$), but this was only temporary.	Neither vitamin D nor exercise contributes to better quality of life, fear of falling or mental wellbeing in community-dwelling healthy older women with sufficient serum 25(OH)D levels.
[34]	The two groups (supplemented with vitamin D and placebo group) were similar in relation to baseline 25(OH)D concentrations. However, at childbirth, the vitamin D group had significantly higher 25-hydroxyvitamin D concentration in comparison to the control group ($p < 0.001$). At baseline, no correlation was observed between 25(OH)D concentration and depression score ($r = 0.13$, $p = 0.09$). There was no significant difference between the two study groups in relation to the baseline depression score. While, the vitamin D group had greater reduction in depression scores than the control group at 38–40 weeks of gestation ($p = 0.01$) also, at 4 and 8 weeks after birth ($p < 0.001$).	Consuming 50 μg vitamin D3 daily during late pregnancy was effective in decreasing perinatal depression levels.
[35]	No evidence of lower depression ($p = 0.339$), lower anxiety ($p = 0.862$), higher flourishing ($p = 0.453$), higher positive moods ($p = 0.518$) or	No evidence of any beneficial effect of monthly vitamin D3 supplementation on mood-related

Ref.	Observation	Conclusion
	lower negative moods ($p = 0.538$) was found in the treatment group (supplemented with vitamin D) compared with the control group at follow-up. Mood outcomes over the study period were similar for the two groups.	outcomes in healthy premenopausal women over the winter period was found, so recommendations for supplementations are not warranted in this population for mood-related outcomes.
[36]	At the end of the study, there were no significant differences in Δ BDI-II score (score at the end of the study minus score at baseline), regardless of analysing all subjects, subjects with or without psycopharmaca, or if performing subgroup analyses based on baseline and final serum 25(OH)D levels combined with categories of baseline BDI-II scores >4 or >13.	The study has not been able to demonstrate any significant effect of vitamin D supplementation on depressive symptoms.
[37]	Levels of 25(OH)D_3 correlated very strongly with energetic arousal (rs = 0.80; $p < 0.05$) and strongly hedonic tone (rs = 0.74; $p < 0.05$) 12 h before the run. There were no significant correlations between levels of 25(OH)D_3 and mood states after the run.	Vitamin D3 supplementation is related to runners' pre-run mood. This effect is nullified when it comes to post-run mood states.
[38]	No relevant differences between the treatment groups were observed regarding depressive symptoms, or any other outcome.	Supplementation with 30 μg per day of vitamin D for 12 months had no effect on depressive symptoms or health related quality of life in older persons with relatively low vitamin D status, clinically relevant depressive symptoms, and poor physical functioning.
[39]	Over time, primary and secondary end points did not differ significantly among the three treatment groups or in subgroups by vitamin D status at baseline. After adjusting for confounders, participants achieving the highest 25(OH)D quartile (Q) at 12 months (44.7-98.9 ng/ml) had the greatest improvements in Mental Component Summary (Q4 = 0.79 vs Q1 = −2.9; $p = 0.03$) and Mental Health scales (Q4 = 2.54 vs Q1 = −3.07; $p = 0.03$); these associations were strongest among participants who were vitamin D deficient at baseline. No association was found for Geriatric Depression Scale ($p = 0.89$).	For mental health, the study suggests no benefit of higher monthly doses of vitamin D3 compared with the standard monthly dose of 600μg. However, achieving higher 25(OH)D levels at 12-month follow-up was associated with a small, clinically uncertain but statistically significant improvement in mental health scores.
[40]	The study showed a decrease in depressive symptoms, while compared with baseline values ($p = 0.026$).	Physical activity and adequate levels of 25(OH)D can be the key factors in maintaining self-reliance in old age.

BDI-II–Beck Depression Inventory–Second Edition; CI–Confidence Interval; OR–Odds Ratio.
